# Novel non-resonant, low-frequency pulse–current circuit for energy-efficient, low-noise transcranial magnetic stimulation

**DOI:** 10.3389/fnins.2025.1610764

**Published:** 2025-06-18

**Authors:** Xinhua Tan, Zongrui Tian, Jiasheng Tian, Yingwei Li, Jian Shi

**Affiliations:** ^1^School of Information Science and Engineering, Yanshan University, Qinhuangdao, China; ^2^School of Electronic Information and Communications, Huazhong University of Science and Technology, Wuhan, Hubei, China; ^3^Key Laboratory of Space Ocean Remote Sensing and Application, Ministry of Natural Resources, Beijing, China; ^4^Hebei Key Laboratory of Information Transmission and Signal Processing, Qinhuangdao, China

**Keywords:** transcranial magnetic stimulation, triangle pulse–current waveform, non-high-frequency circuit, low energy consumption, low noise

## Abstract

**Introduction:**

Transcranial magnetic stimulation (TMS) is increasingly used for non-invasive neuronal activation. By harnessing a pulsed magnetic field, TMS induces electric currents that target the central nervous system. However, its efficacy is often limited by two critical challenges: excessive heat generation and the loud “clicking” noise produced by rapid coil pulsing. These limitations reduce both performance and patient comfort, hindering broader clinical adoption. To overcome these challenges, this study proposes a novel circuit architecture.

**Materials and methods:**

First, the principle of the triangular pulse–current waveform and its sensitivities were studied. The relationships between the waveform parameters and the induced electric field in the human brain were explored to ensure the necessary depolarization of the nerve membrane potential. Subsequently, theoretical analysis, calculations, and a particle swarm optimization algorithm were employed to optimize the pulse–current waveform. The aim was to substantially reduce both the clicking noise (vibration energy) and the ohmic heat generated by the TMS coil. As a result, three typical optimized triangular pulse–current waveforms were obtained under three distinct conditions. Finally, based on multi-module cascading and the principles of programmable TMS circuits, a non-resonant, low-frequency switching design and a voltage-dividing system were implemented. The voltage-dividing system—composed of a series resistor and inductor—together with multi-module cascading controlled by pulse-width modulation (PWM) sequences, was used to generate the desired pulse-voltage levels and durations on the TMS coil.

**Results:**

Three variants of non-resonant, low-frequency TMS circuits were implemented based on the optimized pulse–current waveforms. Theoretical expressions for the optimal waveforms, including the IGBT-controlled voltage-dividing system, were presented. Each optimized triangular pulse–current waveform was modeled and simulated in MATLAB Simulink using these expressions. Moreover, by employing a low-frequency PWM controller, high-frequency switching is entirely avoided. The proposed circuit architecture, which combines a finite series of cascaded modules with the voltage-dividing network, can reproduce any of the optimized pulse–current waveforms as required.

## 1 Introduction

Transcranial magnetic stimulation (TMS) is a non-invasive technology utilized to stimulate and modulate neurons in the human brain. It is extensively used in the treatment of major depressive disorder, Parkinson's disease, post-traumatic stress disorder, and acute ischemic stroke, as evidenced by the references (Harel et al., 2014; Spagnolo et al., 2021; Petrosino et al., 2021). In 1980, Merton and Morton introduced a high-voltage stimulator, known as transcranial electrical stimulation, capable of activating the cerebral cortex (Merton and Morton, 1980). However, transcranial direct current stimulation often causes discomfort and a tingling sensation at the electrode site. In 1985, Barker introduced a painless and non-invasive technique that has come to be widely recognized as TMS (Baker, 1985). The pulsed current within a TMS coil induces an electric field in the human brain, which acts on the neuronal membrane and depolarizes it. When the strength of the induced electric field reaches the activation threshold, neurons discharge and are fully stimulated (Jin et al., 2012). Over the past four decades, TMS has become an indispensable tool in clinical physiology practices and neuroscience, particularly as an experimental intervention for depression and other psychiatric and neurological disorders (Hallett, 2007; Li et al., 2020; Crowther, 2014). However, TMS devices, especially the TMS coil that operates with brief, high-intensity current pulses reaching up to thousands of amperes, produce substantial amounts of heat and vibration (Hsu et al., 2003), resulting in energy loss and loud clicking noises, which are closely linked to the pulse–current waveforms produced by TMS circuits (Tringali et al., 2012). Currently, the existing TMS devices, especially the pulse circuit generators, provide only a narrow range of choices for the pulse–current waveforms. As a result, the heat and noise produced by TMS coils persist as a significant obstacle, diminishing the effectiveness of TMS treatments in nervous system disorders (Zhang et al., 2023).

Historically, monophasic and biphasic pulse currents were initially put forward with the intention of depolarizing neurons in the cerebral cortex. Their introduction was also aimed at triggering action and motor-evoked potentials within the motor regions of the cerebral cortex. Although monophasic pulses exhibit certain advantages in neural activation (Arai et al., 2007; Goetz et al., 2016; Niehaus et al., 2000; Sommer et al., 2006), biphasic ones are more prevalently employed in clinical settings due to their enhanced effectiveness in treating mental disorders (Goetz et al., 2012a, 2013, 2012b). Moreover, reducing the ohmic loss and the clicking noise generated by the TMS coil is an advantage of the biphasic pulse–current waveform.

In recent decades, numerous researchers (Gattinger et al., 2012; Peterchev et al., 2014; Sorkhabi et al., 2021a, 2022; Li et al., 2022) have focused on optimizing the circuit topology structure to achieve diverse pulse waveforms, improving stimulation effectiveness and flexibility, reducing energy loss, and more. In 2012, Gattinger et al. (2012) introduced a novel repetitive transcranial magnetic stimulation device called FlexTMS. The FlexTMS system, employing a full-bridge circuit with four insulated-gate bipolar transistor (IGBT) modules and an energy storage capacitor, can control the pulse current width, polarity, intensity, and variable-interval dual pulse sequences. However, the circuit system was unsuitable for high-repetition pulses and sensitive to the coil inductance. In 2014, Peterchev et al. (2014) put forward a third-generation controllable pulse parameter device that employed a novel circuit topology with two energy-storage capacitors, enabling more flexible pulse shaping; however, the device also exhibited long decay of the coil current, resulting in larger heat consumption in the TMS coil and IGBT modules. In 2021, Sorkhabi et al. (2021a) introduced the second-generation programmable TMS device, which used cascaded H-bridge inverters along with phase-shifted pulse-width modulation (PWM). Their study (Sorkhabi et al., 2021a) demonstrated that increasing the number of PWM voltage levels from 3 to 5 had a substantial positive impact on both the PWM-based TMS pulse and the changes in membrane voltage. However, no remarkable improvement was observed when further increasing the number of levels from 5 to 7 in a PWM system. Moreover, constructing the device with a 7-level PWM system was complex and entailed high costs. In 2022, Sorkhabi et al. (2022) showed a wider variety of pulse waveforms by utilizing PWM (known as programmable TMS or pTMS). However, the proposed device was unable to present the high stimulation magnitudes. In 2022, Li et al. (2022) introduced a modular pulse synthesizer, which could flexibly produce high-power TMS pulses with user-defined electric field shapes and rapid pulse sequences, maintaining high output quality. In 2022, Zeng et al. (2022) introduced a modular multilevel TMS (MM-TMS) topology composed of 10 cascaded H-bridge modules. The MM-TMS device could generate pulses with up to 21 voltage levels, where the step size could reach up to 1,100 V. This feature enabled the relatively flexible generation of a wide variety of pulse waveforms and sequences. However, the MM-TMS system is intricate, and its control mechanism is convoluted. The increment in voltage steps is discrete, which is limited in clinical practices, and the presence of ultra-short pulses will result in a high switching frequency (Zeng et al., 2022), making the components within the devices susceptible to fatigue and damage (Zeng et al., 2022).

To reduce energy consumption and heat loss in TMS devices, in 2007, Peterchev et al. (2007) proposed a device equipped with a controllable pulse width (PW), which was capable of generating near-rectangular induced electric field pulses. In their research (Peterchev et al., 2007), coil heating was calculated based on the load integral of the coil current, which consumed 2%−34% less energy and experienced 67%−72% less coil heating compared to what was needed for matched conventional cosine pulses. However, the impacts of the pulse shape were not considered. In an effort to decrease the noise associated with TMS coils, in 2014, Goetz et al. (2014a) proposed a redesign of both the pulse waveform and the coil structure, which utilized ultra-brief current pulses (its duration as short as 45 μs) to power a prototype coil, leading to a reduction in the peak sound pressure level by over 25 dB when compared with the traditional TMS ones. Moreover, they presented an enhanced mechanical configuration that could suppress sound at its origin and prevent the transmission of residual sound to the coil surface.

Nevertheless, Goetz et al. (2014a) failed to account for the effects of the shape of the current pulse on the sound or noise. Owing to electromagnetic forces, the coil emits clicking sounds, which pose a potential risk of causing hearing impairment (Dhamne et al., 2014; Tringali et al., 2012). Simultaneously, due to the nonlinearity and the multitude of interactions within the brain, these clicking noises have the potential to compromise the efficacy of TMS (Goetz et al., 2014b; Siebner et al., 1999). Moreover, the noise might make the patient restless, agitated, or anxious, which could have a substantial and detrimental effect on the therapeutic outcome (Dhamne et al., 2014).

Currently, conventional pulse–current waveforms, such as traditional sine or symmetric pulse waveforms, have been extensively studied and modeled to reduce energy consumption and clicking noise. Optimization algorithms such as particle swarm optimization and genetic algorithms have been employed to derive optimized waveforms within restricted conditions (Zhang et al., 2023). From both theoretical and electromagnetic simulation standpoints, a large number of optimized waveforms have been proposed, but it is difficult for the existing pulse–current circuit structures and designs to meet these proposed pulse–current waveforms. Additionally, the current TMS drive system, which relies on a resistor/capacitor-inductor circuit for charging and discharging to generate a pulsed current, lacks flexibility and tunability.

Therefore, this research proposes a controlled low-frequency switching structure incorporating multi-modules, each module comprising four Insulated Gate Bipolar Transistors (IGBTs) and a voltage-dividing system. This structure can supply any anticipated voltage levels, thereby remarkably enhancing the flexibility and adjustability of pulse–current waveforms. First, in accordance with the goals of developing a low-energy consumption and low-noise TMS system, the parameters of the desired (or fitted) current waveform in the TMS coil are determined through theoretical calculation and the application of the particle swarm algorithm. Next, a voltage-dividing system is designed, involving the placement of resistors and inductors in series according to the expected current waveform. Finally, PWM compilers generate a sequence of PWM pulse signals to control the switches of the IGBTs, thereby obtaining the desired voltage and realizing the expected pulse–current waveform in the TMS coil.

The proposed controllable low-frequency switching structure differs from the high-frequency ones (Zeng et al., 2022; Sorkhabi et al., 2021b; Nilsson and Riedel, 2010). The obtained pulse–current waveform is much closer to the required current waveform than those of Majid M. S. et al. (Zeng et al., 2022; Nilsson and Riedel, 2010). The innovative aspects primarily lie in the voltage-dividing system and its theoretical expressions, which can compensate for the drawback of only providing discrete voltage levels (Zeng et al., 2022; Sorkhabi et al., 2021b), the programmable PWM compilers, and the multi-module cascading technology.

## 2 Materials and methods

### 2.1 Principle of pulse–current waveforms

In this section, the expressions of the TMS pulse–current waveform are given. Numerous studies have demonstrated that the biphasic triangular stimulation waveform can generate an induced electric field that is close to a rectangular shape in the human brain, and its efficiency in stimulating brain neurons is substantially higher than that of traditional pulse shapes, such as the biphasic sine waveform (Goetz et al., 2016, 2013; Peterchev et al., 2014, 2007). Therefore, based on the principle of the TMS circuit, this study constructs the functional relationship of the biphasic triangular stimulation waveform in accordance with the TMS requirements and establishes a parametric model by which the impacts of the pulse–current waveform shape on the TMS coil ohmic loss and its experienced impulse of the electromagnetic force are analyzed. In contrast, all stimulation effectiveness on neurons in the human brain is kept unchanged.

The generated pulse–current waveform can be expressed as a three-segment function based on the TMS process and its objective. The first segment (0~*t*_1_) represents the reverse charging process with an initial current *I*_01_ of 0 A, where four series of PWM sequences operate the switches of the four IGBTs to cause the TMS coil current to increase in the negative direction for obtaining the requested current waveform. Supposing the voltage exerted upon the TMS coil at each segment of the pulse–current waveform is constant, the first segment of the pulse–current waveform can be given as follows:


(1) $$\begin{array}{l} i_{1}=-\frac{m_{1}V_{0}}{R}\left(1-e^{-\frac{R}{L}t}\right),t\leq t_{1}, \end{array}$$

where *R* and *L* are the resistor and inductor of the TMS coil, *m*_1_ is constant, and -*m*_1_*V*_0_ is the voltage experienced by the TMS coil at the first segment. The second segment involves applying a forward voltage to the TMS coil at an initial current of *I*_02_, causing the coil to discharge first and then charge forward until the positive peak current is reached at *t* = *t*_1_+*t*_2_. Similarly, the second segment of the pulse–current waveform is controlled by programmable PWM pulses, during which the current changes from its maximum negative value to its maximum positive value. During this process, an electric field is induced in the human brain, depolarizing the cell membrane potential. The electric current in the second segment can be expressed as


(2) $$\begin{array}{l} i_{2}=\frac{m_{2}V_{0}}{R}\left(1-e^{-\frac{R}{L}\left(t-t_{1}\right)}\right)+I_{02}e^{-\frac{R}{L}\left(t-t_{1}\right)},t_{1}\leq t\leq t_{1}+t_{2}, \end{array}$$

where *m*_2_ is constant, and *m*_2_*V*_0_ is the voltage experienced by the TMS coil at the second segment. *t*_1_ represents the end of the first segment, and *I*_02_ represents the electric current of the TMS coil at *t* = *t*_1_. In the third segment, a reverse voltage is applied to the TMS coil at an initial current *I*_03_, causing a rapid discharge to reduce energy consumption. During the third process, the electric current decreases rapidly to zero via the discharging of the TMS coil. The electric current in the TMS coil can be expressed as


(3) $$\begin{array}{ll} i_{3}=-\frac{m_{3}V_{0}}{R}\left(1-e^{-\frac{R}{L}\left(t-t_{1}-t_{2}\right)}\right)+I_{03}e^{-\frac{R}{L}\left(t-t_{1}-t_{2}\right)}, & t_{1}+t_{2}\leq t\leq t_{1}\\ \;+t_{2}+t_{3}, \end{array}$$

where *t*_2_ represents the time spent on the second segment, *I*_03_ represents the electric current of the TMS coil at *t* = *t*_1_ + *t*_2_, *m*_3_ is constant, *and*−*m*_3_*V*_0_ is the voltage experienced by the TMS coil at the third segment. At the third segment, the current in the TMS coil becomes zero at *t* = *t*_3_.

### 2.2 Optimization of pulse current waveform

#### 2.2.1 The sensitivity of pulse current waveform

**Figure 1:**
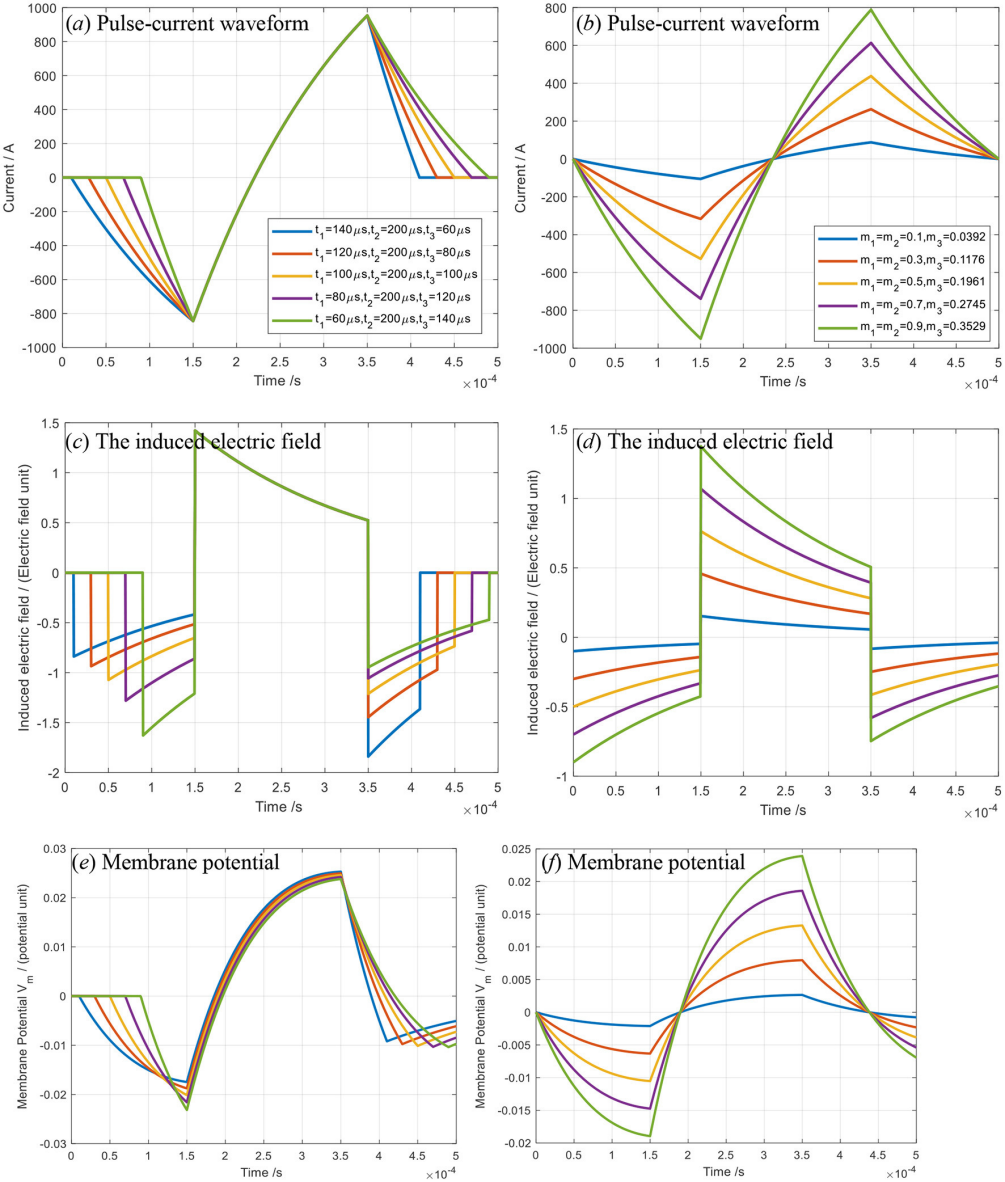
The pulse–current waveform **(a, b)**, induced electric field **(c, d)** and membrane potential of neurons **(e, f)**: **(a, c, e)** for variable pulse-current width *t*1 and *t*3; **(b, d, f)** for variable slope but invariable time of each segment.

In terms of Equations 1–3, supposing *m*_1_*V*_0_ or *m*_2_*V*_0_ = (100V, 120V, 140V, 160V, 180V) and *m*_3_*V*_0_ = (45V, 65V, 85V, 105V, 125V), *t*_1_=*t*_3_ = (60us,80us,100us,120us,140us) and *t*_2_ = 200us, we can obtain the pulse current waveforms in the TMS coil, as plotted in Figure 1. In Figure 1, the internal resistance *R* and inductance *L* of the TMS coil are *R* = 0.05 Ω and *L* = 20 μH (Peterchev et al., 2014; Zeng et al., 2022), respectively. According to Maxwell's equations, the induced electric field in the human brain is proportional to the time derivative of the current waveform, where the proportionality coefficient between the induced electric field and the pulse current is related to the physical properties of the human brain medium and the TMS system. The induced electric field *E*_*m*_(*t*) acts on neuron cells, prompting a change in the membrane potential *V*_*m*_(*t*) of neurons. The relationships between *E*_*m*_(*t*) and *V*_*m*_(*t*) can be given as


(4) $$\begin{array}{l} kE_{in}\left(t\right)=\frac{V_{m}\left(t\right)}{R_{m}}+C_{m}\frac{dV_{m}\left(t\right)}{dt}, \end{array}$$

where *k* is the constant related to the effective conductivity of neuron cells, and *R*_*m*_ and *C*_*m*_ are the effective resistance and capacitance of neurons, respectively.

In Figure 1a, the negative peak magnitude of the first segment pulse current remains unchanged, and the slope of the dispersive relationship curve increases negatively with the pulse width *t*_1_ decreasing, where it is evident that *m*_1_*V*_0_ or *m*_3_*V*_0_ increases with *t*_1_ or *t*_3_ decreasing. Similarly, the slope of the first segment curve increases negatively with *m*_1_*V*_0_ increasing when *t*_1_=constant, as shown in Figure 1b. The induced electric field magnitude is closely related to the slope of the first segment of the dispersive curve, as shown in Figure 1c. For the second segment, the induced electric field magnitude in the human brain is closely related to the slope of the second segment dispersive curve. It increases as the slope of the second segment of the dispersive curve increases, as shown in Figure 1d.

In Figure 1a, the slope of the second segment of the dispersive curve is kept unchanged, resulting in an induced electric field in the human brain that maintains a stable, approximately rectangular shape. For the third segment, the dispersive behavior mirrors that of the first or second segment. The slopes of the first, second, and third segments are approximately -*m*_1_*V*_0_/*L*, *m*_2_*V*_0_/*L*, and -*m*_3_*V*_0_/*L*, respectively, due to the short durations of times *t*_1_, *t*_2_, *andt*_3_. Consequently, the shape of the triangular pulse–current waveform can be controlled by the voltages -*m*_1_*V*_0_, *m*_2_*V*_0_, and -*m*_3_*V*_0_ (or equivalently, by the parameters -*m*_1_, *m*_2_, and -*m*_3_), as well as by the pulse–current widths *t*_1_, *t*_2_, and *t*_3_. The membrane potential *V*_*m*_(*t*) of neurons is calculated using Equation 4, as shown in Figures 1e, f.

**Figure 2:**
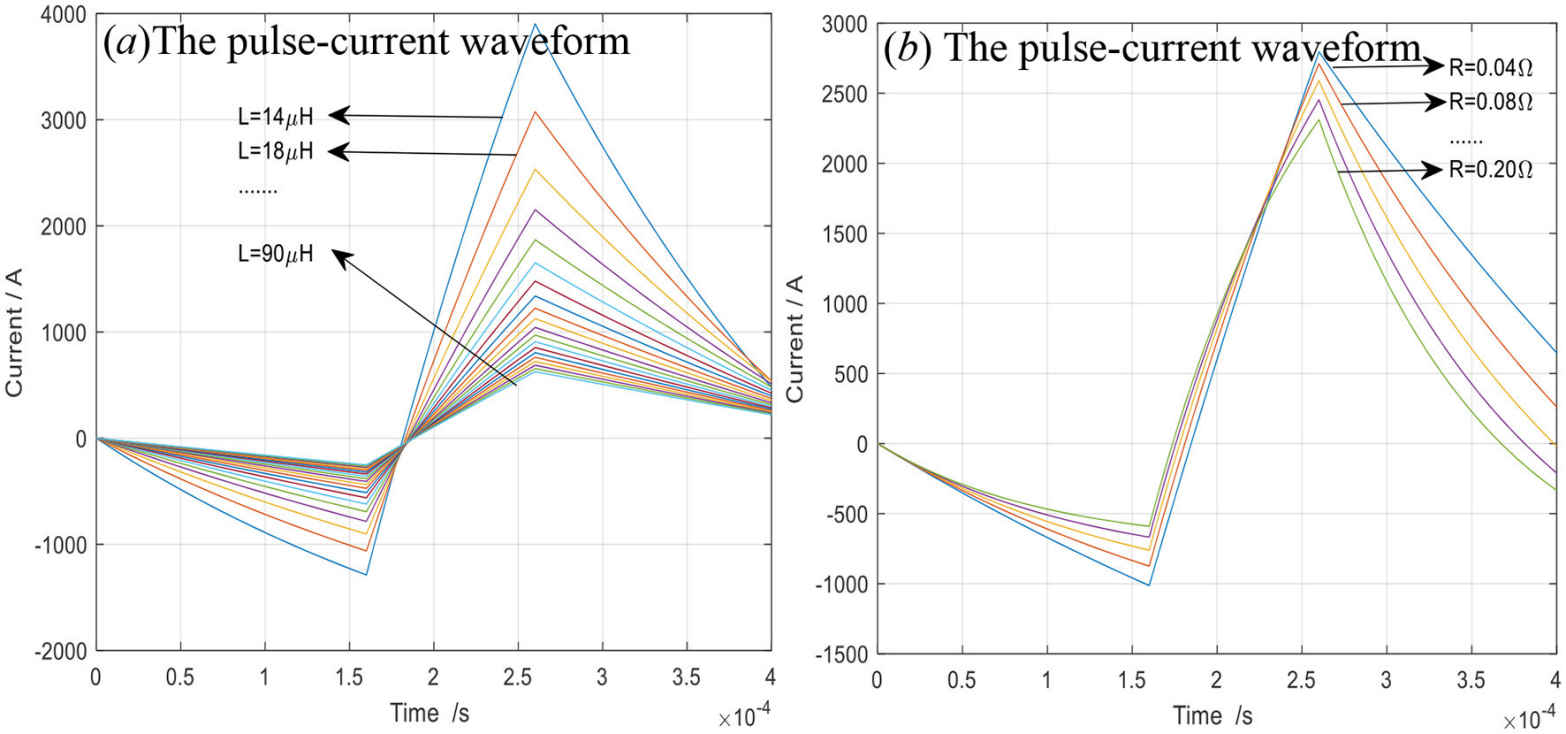
The pulse–current waveform varying with the resistance and inductance of TMS coil **(a)** for variable inductances of TMS coil; **(b)** for variable resistances of TMS coil.

In Figure 2, the pulse–current waveform varies with the resistance *R* and inductor *L*, whose peak magnitude decreases with *R* and *L* increasing, and the response slope (d*i*/d*t*) reduces. In Figure 2a, the peak magnitude of the pulse–current waveform decreases with the TMS coil inductance *L* increasing, resulting in the slope magnitude of the pulse–current waveform decreasing. In Figure 2b, the peak magnitude of the pulse–current waveform decreases with the TMS coil resistance *R* increasing, resulting in the slope magnitude of the pulse–current waveform decreasing. Since the TMS coil is fixed, *R* and *L* remain constant and are excluded from further optimization in this study.

#### 2.2.2 Calculation of energy consumption and clicking noise

To maintain the TMS coil's effectiveness, we optimize the triangle pulse–current waveform to reduce the TMS coil noise (clicking) and its ohmic loss. Two indicators, the ohmic loss *Q* and coil electromagnetic force impulse *P*, were used to describe the performance of the excited pulse–current waveform in reducing the TMS coil noise and its ohmic loss. According to the principle of the TMS coil ohmic loss *Q*, the generated *Q* of the TMS coil with resistance *R*, due to a pulse current *i*(*t*) (in one period *T*), can be expressed as follows:


(5) $$\begin{array}{l} Q=\int_{0}^{T}i^{2}\left(t\right)Rdt. \end{array}$$

Additionally, the magnetic force experienced by current-carrying coil 1 is exerted by TMS coil 2 as follows:


(6) $$\begin{array}{l} \overset{⇀}{F}=\frac{\mu_{0}}{4\pi }\oint_{c1}I_{1}\overset{⇀}{dl_{1}}\times \oint_{c2}\frac{I_{2}d\overset{⇀}{l_{2}}\times \overset{⇀}{R_{21}}}{R_{21}^{3}} \end{array}$$

where ${\overset{⇀}{R}}_{21}$ is the distance vector from the current element $I_{2}d{\overset{⇀}{l}}_{2}$ to the current element $I_{1}d{\overset{⇀}{l}}_{1}$, which is proportional to the square of the coil current *I* = *I*_1_ = *I*_2_ = *i*(*t*) owing to the series coils, that is $\overset{⇀}{F}\;\propto \;I^{2}$.

Because the magnetic force impulse $\overset{⇀}{P}$ experienced by the TMS coil is an integral of $\overset{⇀}{F}$ over one time period *T*, the value of $\overset{⇀}{P}$ is also proportional to *i*^2^(*t*):


(7) $$\begin{array}{l} \overset{⇀}{P}=\int_{0}^{T}\overset{⇀}{F}dt\propto \int_{0}^{T}i^{2}\left(t\right)dt \end{array}$$

As shown in the aforementioned equations, *Q* and $\overset{⇀}{P}$ are integrals of *i*^2^(*t*) throughout the pulse current in the TMS coil, and thus, *Q* and $\overset{⇀}{P}$ can reach their minimum values simultaneously.

#### 2.2.3 Optimization of the pulse current waveform

In this section, taking into account reducing the energy consumption and the clicking noises generated by the TMS coil, the pulse–current waveform is optimized.

The pulse duration is very short, exp(-*R*/L^*^t)≈1-*R*/L^*^t, and thus Equations 1–3 can be expressed by three linear ones as follows:


(8) $$\begin{array}{l} i_{1}=-k_{1}t,t\leq t_{1} \end{array}$$

(9) $$\begin{array}{l} i_{2}=k_{2}t+b_{2},t_{1}\leq t\leq t_{1}+t_{2} \end{array}$$

(10) $$\begin{array}{l} i_{3}=-k_{3}t+b_{3},t_{1}+t_{2}\leq t\leq t_{1}+t_{2}+t_{3} \end{array}$$

The second segment, current *i*_2_, mainly generates the induced electric field, whose slope *k*_2_ will define the amplitude of the induced electric field in the human brain within the time *t*_2_. In order to reduce the energy loss in the third segment current *i*_3_, the slope *k*_3_ of the current in the third section should be as large as possible for the fastest decline. Obviously, *k*_1_, *k*_2_, and *k*_3_ are mainly related to *m*_1_*V*_0_/*L*, *m*_2_*V*_0_/*L* and *m*_3_*V*_0_/*L*, respectively. If *V*_0_ is the largest voltage magnitude presented by the TMS source, then *m*_2_=1. The optimization of the pulse–current waveform of a given TMS coil mainly focuses on finding the slope *k*_1_ and the time *t*_1_ of the first section of the waveform. Of course, the pulse current period *T*=*t*_1_ +*t*_2_ +*t*_3_ is given based on the TMS system. Therefore, by substituting Equations 1–3 or 8–10 into Equations 5–7, we can find out the slope *k*_1_ and the time *t*_1_ when *Q* or *P* reaches the minimum.

**Figure 3:**
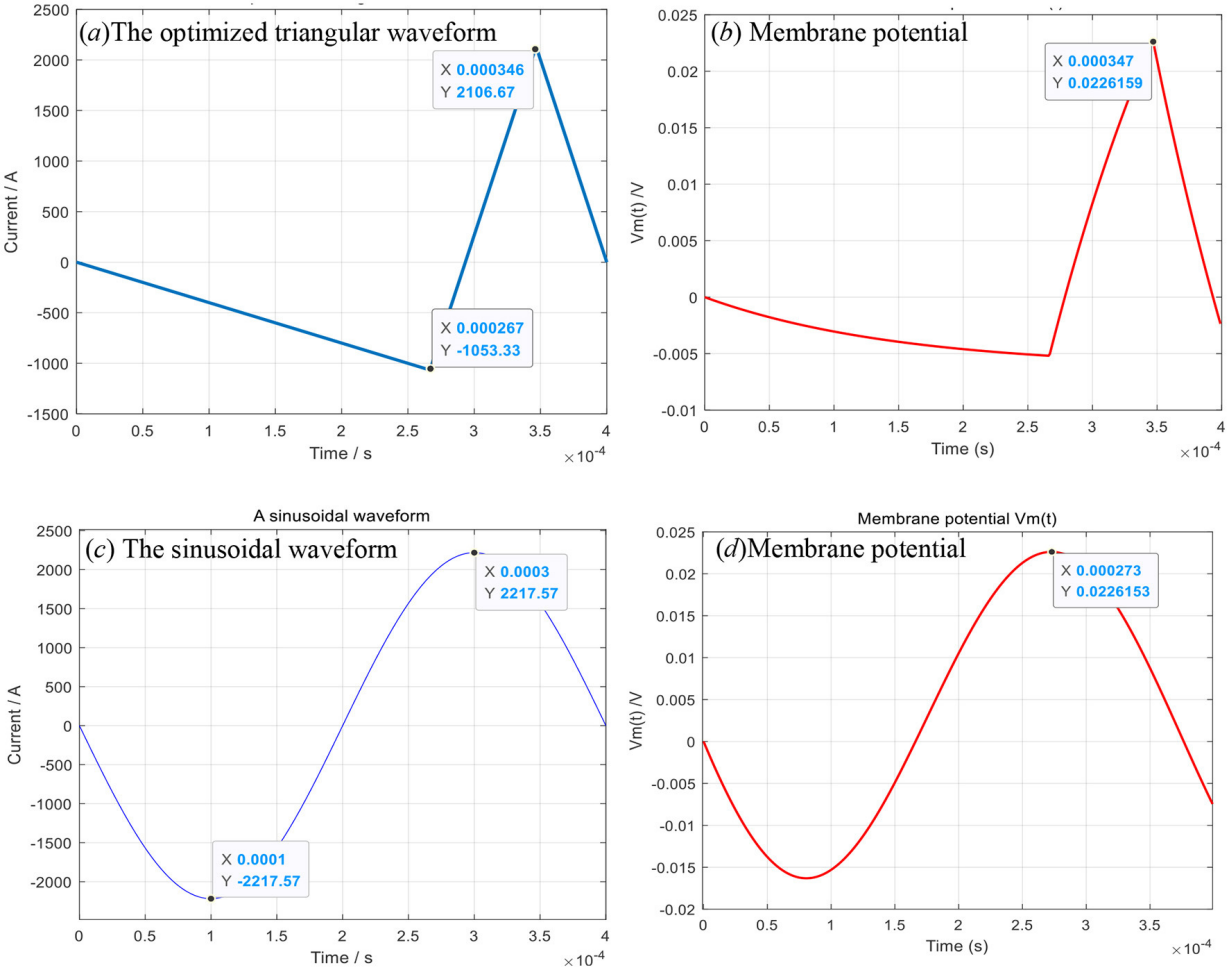
The triangular **(a)**/sinusoidal **(c)** waveform and the corresponding membrane potentials **(b, d)**.

**Figure 4:**
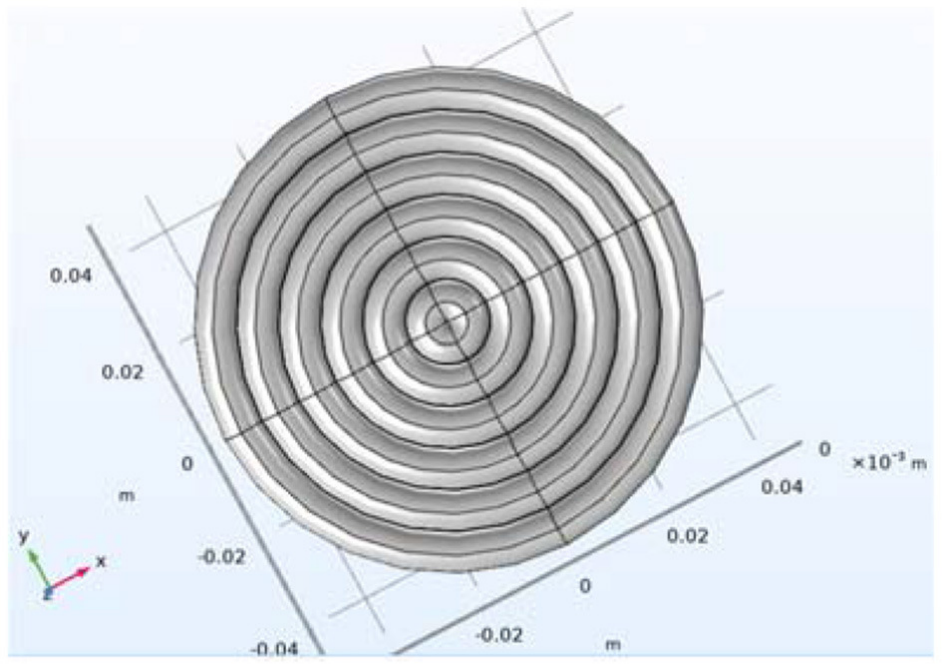
The model of the six-turn coil.

Under the premise of maintaining a good stimulation effect, the triangular waveform has less heat loss and noise compared to the sinusoidal waveform. The membrane potential (22.6159 mV) produced by the optimized triangular waveform in the human brain, shown in Figures 3a, b, is the same as the one (22.6153 mV) produced by the sinusoidal waveform, shown in Figures 3c, d. However, the ohmic heat loss (Q = 13.6527J, *R* = 0.05Ω) produced by the triangular waveform is less than the ohmic loss (Q = 49.1764J) produced by the sinusoidal waveform. When the optimized current is exerted upon the six turns of coils shown in Figure 4, the impulses experienced by the six turns of coils (radius = 0.004, 0.012,0.02,0.028,0.036,0.044 m) are 0.0018, 0.0029, 0.0032, 0.0028, 0.0017 and −0.001N^*^s, respectively, and are less than those impulses (*P* = 0.0065, 0.0104, 0.0115, 0.0102, 0.0062, −0.0036N^*^s) experienced by the six coils when the sinusoidal current is exerted upon the six coils.

(1) The optimized pulse–current waveform I

**Figure 5:**
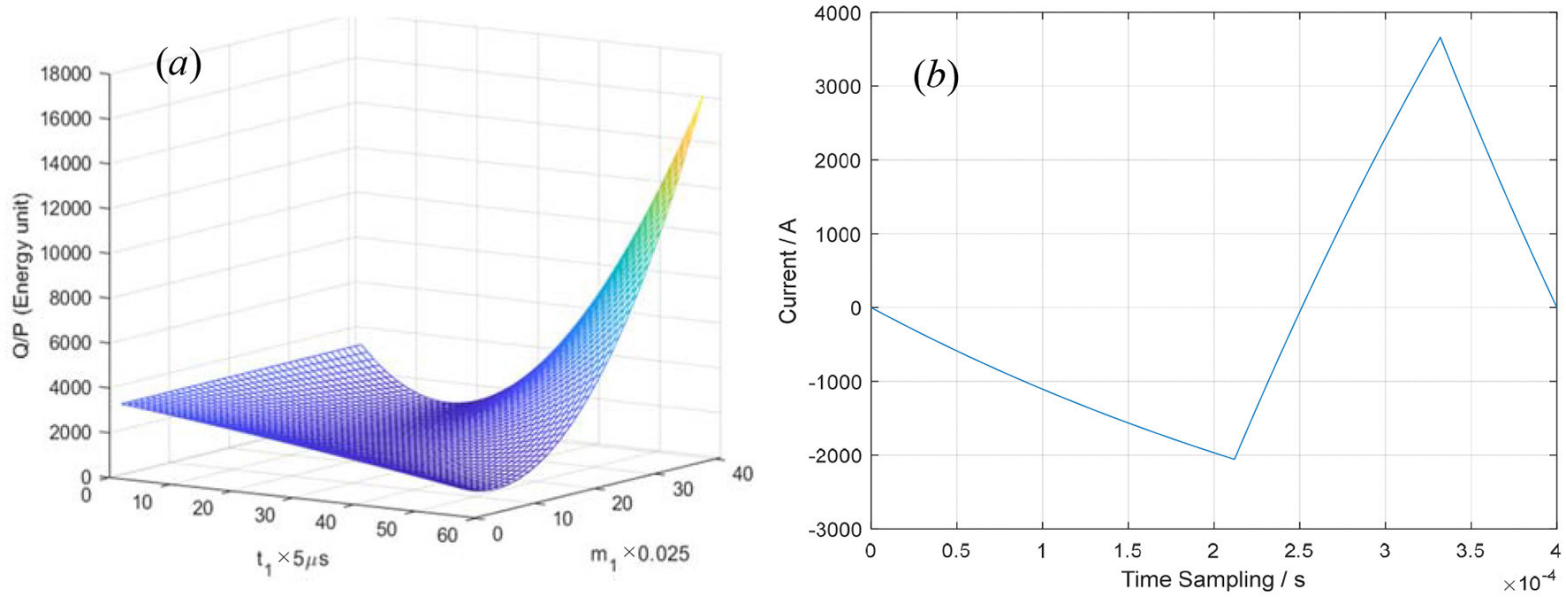
The integral of *i*^2^(*t*) over one period **(a)** and the optimized pulse–current waveform **(b)**.

Supposing *T* = 400 μs, *t*_2_ = 120 μs, *V*_0_ = 1,000 V, *R* = 0.05Ω, and L = 20 μH, the values of *Q* and/can also be calculated using Equations 5, 7 by inputting the pulse current *i*(*t*) expressed by Equations 1–3, as shown in Figure 5. It is obvious that *Q* or |$\overset{⇀}{P}$| appears to be a saddle-shaped function of *m*_1_ and *t*_1_. In Figure 5a, the integral of *i*^2^(*t*) over the whole period T = 400 μs is shown when *t*_1_ = 0–280 μs and *m*_1_ = 0–1. When *m*_3_ = 0.959684 ≈ 1, the minimum of *Q* or |$\overset{⇀}{P}$| can be obtained by using numerical calculation and particle swarm optimization, and the solutions by the two algorithms are in good agreement. The pulse–current waveform is approximately given as follows:


(11) $$\begin{array}{l} i_{1}=-\frac{0.25V_{0}}{R}\left(1-e^{-\frac{R}{L}t}\right),t\leq t_{1} \end{array}$$

(12) $$\begin{array}{l} i_{2}=\frac{V_{0}}{R}\left(1-e^{-\frac{R}{L}\left(t-t_{1}\right)}\right)+I_{02}e^{-\frac{R}{L}\left(t-t_{1}\right)},t_{1}\leq t\leq t_{1}+t_{2} \end{array}$$

(13) $$\begin{array}{ll} i_{3}=-\frac{V_{0}}{R}\left(1-e^{-\frac{R}{L}\left(t-t_{1}-t_{2}\right)}\right)+I_{03}e^{-\frac{R}{L}\left(t-t_{1}-t_{2}\right)}, & t_{1}+t_{2}\leq t\leq t_{1}\\ \;+t_{2}+t_{3}, \end{array}$$

where *t*_1_ = 210 μs, *m*_1_ = 0.25. In Figure 5b, the optimized pulse–current waveform is shown and governed by Equations 11–13.

(2) The optimized pulse–current waveform II

Similarly, let T=400μs, *t*_2_ = 120 μs, *V*_0_ = 1,000V, *R* = 0.05Ω, L = 20 μH, the values of *Q* or |$\vec{P}$| can be calculated using Equations 5, 7 by inputting the pulse current *i*(*t*) defined by Equations 1–3. When *m*_3_≈1, the minimum of *Q* or |$\vec{P}$| can be obtained through numerical calculation and particle swarm optimization. The results from both algorithms show good agreement. When *t*_2_ = 140 us, the optimized equations can be approximately expressed as follows:


(14) $$\begin{array}{l} i_{1}=-\frac{0.325V_{0}}{R}\left(1-e^{-\frac{R}{L}t}\right),t\leq t_{1} \end{array}$$

(15) $$\begin{array}{l} i_{2}=\frac{V_{0}}{R}\left(1-e^{-\frac{R}{L}\left(t-t_{1}\right)}\right)+I_{02}e^{-\frac{R}{L}\left(t-t_{1}\right)},t_{1}\leq t\leq t_{1}+t_{2} \end{array}$$

(16) $$\begin{array}{ll} i_{3}=-\frac{V_{0}}{R}\left(1-e^{-\frac{R}{L}\left(t-t_{1}-t_{2}\right)}\right)+I_{03}e^{-\frac{R}{L}\left(t-t_{1}-t_{2}\right)}, & t_{1}+t_{2}\leq t\leq t_{1}\\ \;+t_{2}+t_{3} \end{array}$$

**Figure 6:**
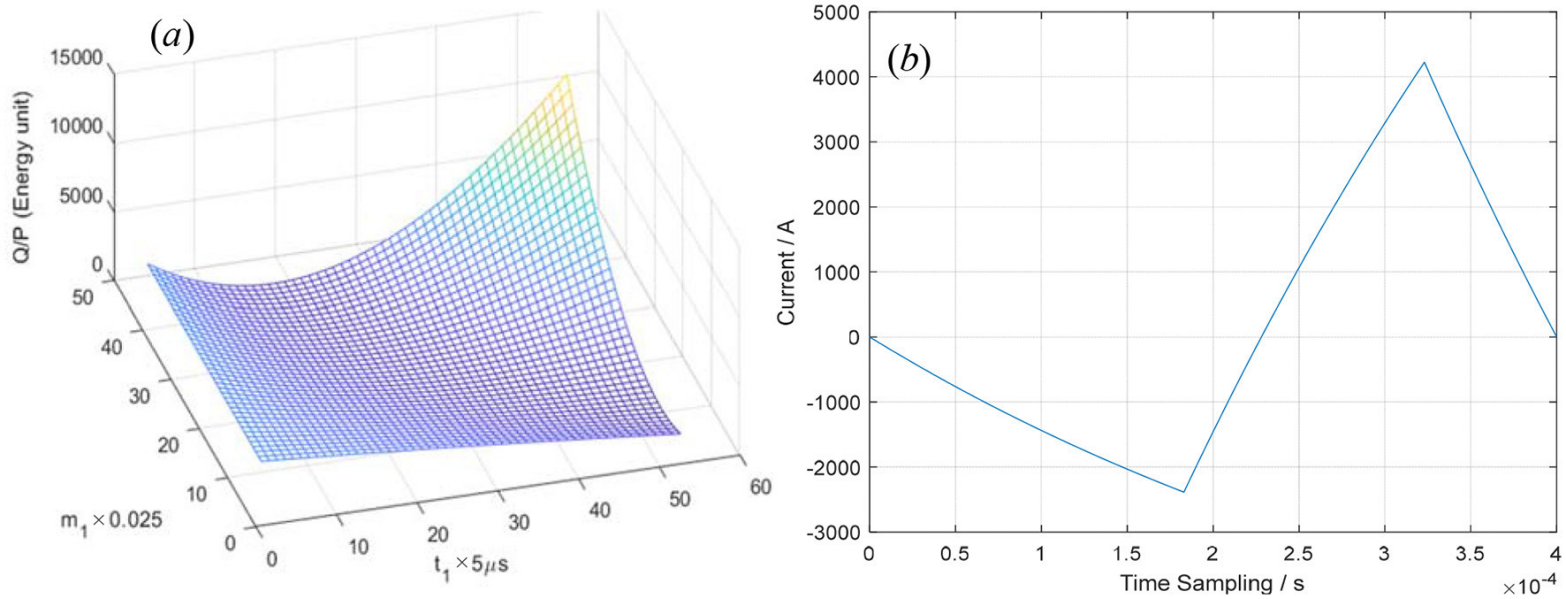
The integral of *i*^2^(*t*) over one period **(a)** and the optimized pulse–current waveform **(b)**.

The optimization process and the optimized waveform are shown in Figure 6. In Figure 6a, the integral of *i*^2^(*t*) over the whole period *T* = 400 μs when *t*_1_ = 0–260 μs and *m*_1_ = 0–1. In Figure 6b, the optimized pulse–current waveform is shown and governed by Equations 14–16, where *T* = 400μs, *t*_1_ = 183μs, *m*_1_ = 0.325, *t*_2_ = 140 μs, *m*_3_ = 0.9951, *V*_0_ = 1,000 V, *R* = 0.05Ω, and *L* = 20 μH.

(3) The optimized pulse–current waveform III

Similarly, when *t*_2_ = 160 us, the optimized equations can be approximately expressed as follows:


(17) $$\begin{array}{l} i_{1}=-\frac{0.47V_{0}}{R}\left(1-e^{-\frac{R}{L}t}\right),t\leq t_{1} \end{array}$$

(18) $$\begin{array}{l} i_{2}=\frac{V_{0}}{R}\left(1-e^{-\frac{R}{L}\left(t-t_{1}\right)}\right)+I_{02}e^{-\frac{R}{L}\left(t-t_{1}\right)},t_{1}\leq t\leq t_{1}+t_{2} \end{array}$$

(19) $$\begin{array}{ll} i_{3}=-\frac{V_{0}}{R}\left(1-e^{-\frac{R}{L}\left(t-t_{1}-t_{2}\right)}\right)+I_{03}e^{-\frac{R}{L}\left(t-t_{1}-t_{2}\right)}, & t_{1}+t_{2}\leq t\leq t_{1}\\ \;+t_{2}+t_{3} \end{array}$$

**Figure 7:**
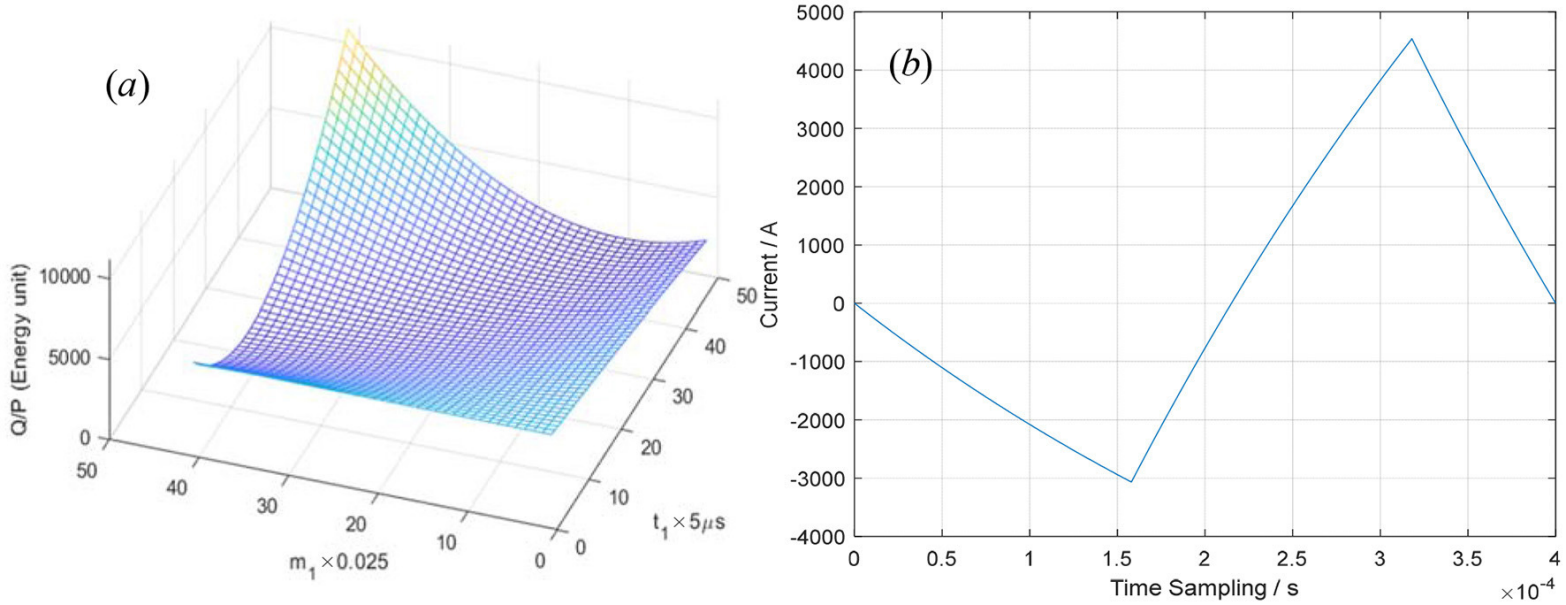
The integral of *i*^2^(*t*) over one period **(a)** and the optimized pulse–current waveform **(b)**.

The optimization process and the optimized waveform are shown in Figure 7. In Figure 7a, the integral of *i*^2^(*t*) over the whole period *T* = 400 μs when *t*_1_ = 0–240μs and *m*_1_ = 0–1. In Figure 7b, the optimized pulse–current waveform is shown and defined by Equations 14–16, where *T* = 400 μs, *t*_1_ = 158 μs, *m*_1_ = 0.47, *t*_2_ = 160 μs, *m*_3_ = 0.9971, *V*_0_ = 1,000V, *R* = 0.05 Ω, and *L* = 20 μH.

Generally, treatment durations vary significantly depending on the type of disease. According to the theoretical analysis and calculations, the article presented three optimized waveforms (I, II, III) under three different treatment periods (*t*_2_ = 120 μs, 140 μs, and 160 μs), as described above. In the following section, the circuits will be meticulously designed to effectively implement these three optimized waveforms, aiming to bring them into practical applications.

### 2.3 Circuit model

**Figure 8:**
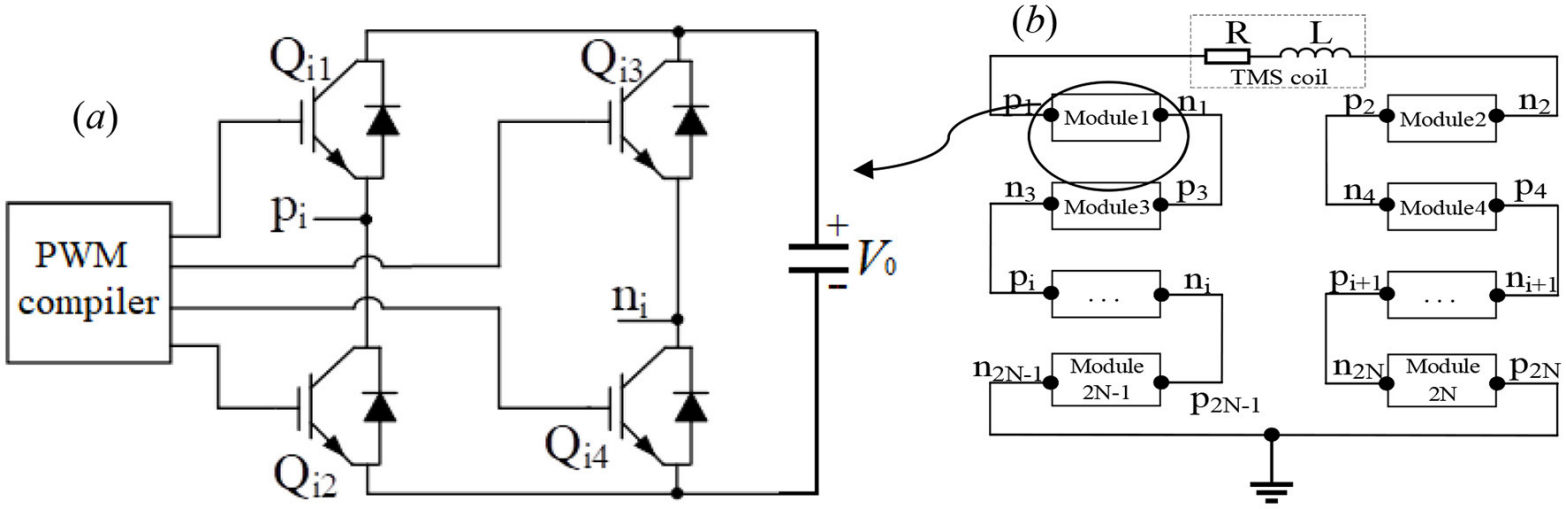
The four IGBT modules **(a)** and 2N modules are connected in series **(b)**.

The study presents the programmable transcranial magnetic stimulation (pTMS) system, which controls the on and off states of the four insulated gate bipolar transistors (IGBTs) using pulse width modulation (PWM). A controlled module is made of four IGBTs on the H-bridge circuit, connected with the direct current (DC) capacitor, which is the large electric field energy storage bank and can be used as a constant voltage source *V*_0_. The PWM voltage signals control the four IGBTs on the H-bridge so that they work together to obtain the required voltage and current on the TMS coil, resulting in a time-varying magnetic field generated by the current-carrying TMS coil. The four-IGBT module can provide three voltage levels with *V*_0_, –*V*_0_, and 0 using PWM voltage signals, which are controlled by a programmable compiler, as shown in Figure 8a. In Figure 8a, the pulse current originates from the capacitor with the constant voltage *V*_0_ to Q_i1_ and then passes through p_i_ to the TMS coil before returning to *n*_i_ to finally pass through Q_i4_ to the capacitor, where IGBT Q_i1_ and IGBT Q_i4_ are the on states, while IGBT Q_i2_ and IGBT Q_i3_ are the off states. This working mode (M1) exerts a positive voltage *V*_0_ upon the TMS coil. When IGBT Q_i1_ and IGBT Q_i4_ are in the off states, and IGBT Q_i2_ and IGBT Q_i3_ are in the on states, the pulse current flows from *n*_i_ through the TMS coil and returns via node p_i_, passing through Q_i2_ back to the capacitor. This configuration, referred to as working mode M2, applies a negative voltage of -*V*_0_ to the TMS coil. When only Q_i1_ or Q_i3_ is in the on state while the others are off, the H-bridge outputs zero voltage ±0, corresponding to working mode M0.

In traditional circuits, generating three or more voltage levels is typically required to produce the desired pulse–current waveform. Recently, numerous scholars have employed multi-modular H-bridge superposition and high-frequency switching to achieve this. However, TMS circuit systems utilizing high-frequency switches face two major drawbacks: (1) The switching period of high-frequency devices generally ranges from several to tens of microseconds. Such frequent switching increases the risk of device damage. (2) While multi-module IGBT cascading can produce several voltage levels, these levels are discrete and follow fixed multiples (for example, 0, ±*V*_0_, ±2*V*_0_, ±3*V*_0_,…). This limitation prevents the generation of arbitrary voltage levels, significantly restricting practical applications, as shown in Figure 8b. For example, the above-optimized pulse–current waveforms II and III cannot be accurately generated using the multi-module system shown in Figure 8b, as doing so would require dozens or even hundreds of H-bridge modules connected in series.

In this study, a voltage-dividing system composed of a resistor and an inductor is connected in series to the TMS coil circuit. This voltage-dividing system does not alter the pulse–current waveform shape and can provide any arbitrary required voltage levels, effectively reducing the number of modules connected in series.

#### 2.3.1 Circuit model for the optimized pulse–current waveform I

**Figure 9:**
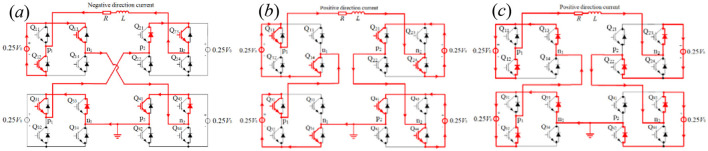
0.25*V*_0_ exerted upon the TMS coil **(a)** and ±*V*_0_ exerted upon the TMS coil **(b, c)**.

In terms of Equation 11, *m*_1_ = 0.25 and *m*_3_ ≈ 1 illustrate that −0.25*V*_0_ is exerted upon the TMS coil at *t* ≤ *t*_1_=210μs (shown in Figure 9a), *V*_0_ is exerted upon the TMS coil at *t*_1_=210μs < *t* ≤ *t*_1_+ *t*_2_=330μs (shown in Figure 9b), and –*V*_0_ is exerted upon the TMS coil at *t* > *t*_1_+ *t*_2_ = 330 μs(shown in Figure 9c). For the optimized pulse–current waveform I, four modules are connected in series at the first segment, with only 1 module providing a voltage of −0.25*V*_0_ in working mode M2 and the other three modules providing a voltage of 0V in working mode M0, as shown in Figure 9a. At the second segment of the pulse current waveform, four modules are connected in series, with each module providing a voltage of 0.25 *V*_0_ in working mode M1, as shown in Figure 9b. At the third segment of the pulse current waveform, four modules are connected in series, with each module providing a voltage of 0.25 *V*_0_ in working mode M2, as shown in Figure 9c. The red lines demonstrate the direction of the electric current flow and the states of the switches in Figure 9.

**Figure 10:**
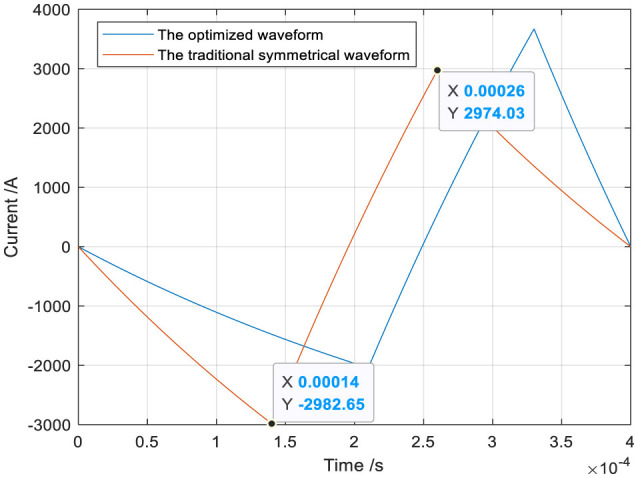
The optimized and traditional symmetrical triangular waveform.

The optimized waveform I and the traditional symmetrical triangular waveform are shown in Figure 10. The heat loss Q can be calculated by substituting the optimized waveform current and the traditional symmetrical triangular current into Equation 5. The ohmic loss Q (53.3994J) produced by the optimized waveform is lower than that (59.3301J) produced by the traditional symmetrical waveform, and the heat loss decreases by 10%.

#### 2.3.2 Circuit model for the optimized pulse–current waveform II

**Figure 11:**
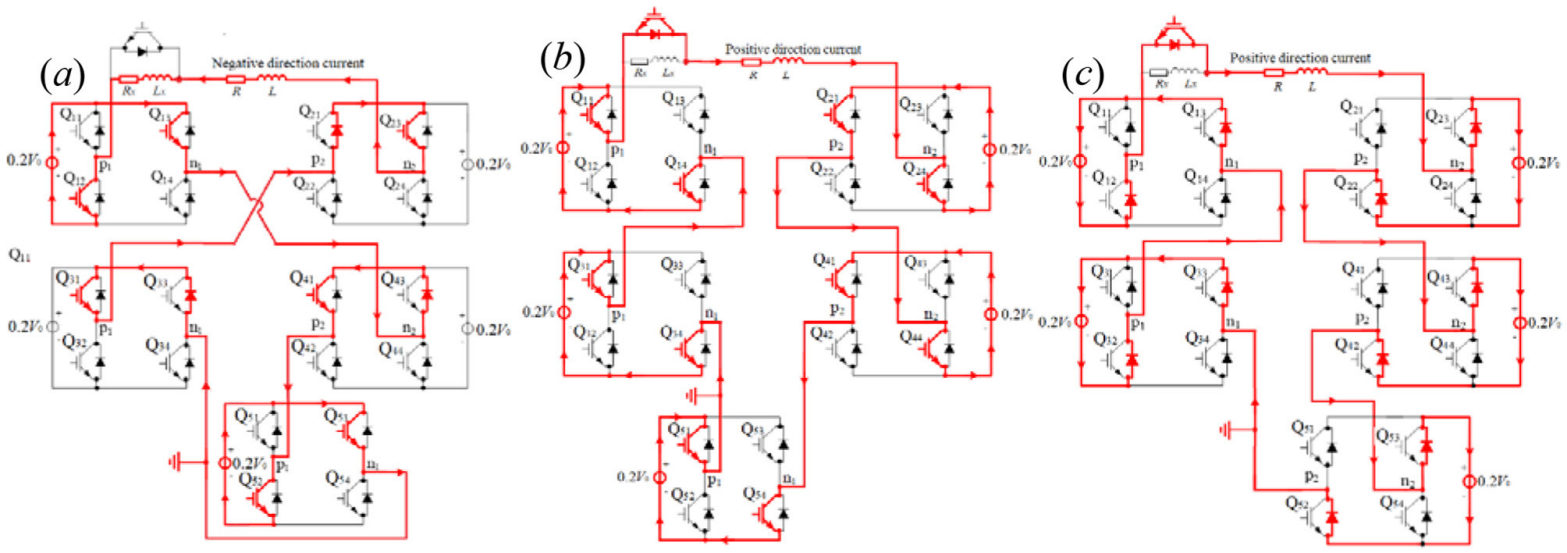
0.325*V*_0_ exerted upon the TMS coil **(a)** and ±*V*_0_ exerted upon the TMS coil **(b, c)**.

According to Equation 14, *m*_1_ = 0.325 and *m*_3_ ≈ 1 illustrate that −0.325*V*_0_ is exerted upon the TMS coil at *t* ≤ *t*_1_ = 183 μs (shown in Figure 8a), *V*_0_ is exerted upon the TMS coil at *t*_1_ = 183 μs < *t* ≤ *t*_1_ + *t*_2_ = 323 μs (shown in Figure 11b), –*V*_0_ is exerted upon the TMS coil at *t* > *t*_1_ + *t*_2_ = 323 μs(shown in Figure 11c). In other words, for the optimized pulse–current waveform II, five modules are connected in series at the first segment, with only two modules providing a voltage of −0.4*V*_0_ in working mode M2 and other three modules providing a voltage of 0V in working mode M0, as shown in Figure 11a, where the voltage-dividing system (composed of resistance *R*_*x*_ = 3*R*/13 and inductor *L*_*x*_= 3*L*/13) is experienced by −0.075 *V*_0_. At the second segment of the pulse current waveform, five modules are connected in series, with each module providing a voltage of 0.2 *V*_0_ in working mode M1, as shown in Figure 11b. At the third segment of the pulse current waveform, five modules are connected in series, with each module providing a voltage of −0.2*V*_0_ in working mode M2, as shown in Figure 11c. The voltage-dividing system is controlled by an IGBT, which is in the off state during the first segment of the triangle pulse current (*t* ≤ *t*_1_ = 183 μs) and is in the on state at the other two segments (*t* > *t*_1_=183 μs). At *t* ≤ *t*_1_ = 183 μs, the negative current passes through the voltage-dividing system because the IGBT connected in parallel with it is in the off state, while the negative or positive current passes through the IGBT connected in parallel with the voltage-dividing system at the second and third segments of the pulse–current at *t* > *t*_1_ = 183μs because the IGBT is in the on state. Equation 14 can be expressed by the following:


(14a) $$\begin{array}{l} i_{1}=-\frac{0.325V_{0}}{R}\left(1-e^{-\frac{R}{L}t}\right)\text{=}-\frac{0.4V_{0}}{R\text{+}R_{x}}\left(1-e^{-\frac{R+R_{x}}{L+L_{x}}t}\right),t\leq t_{1} \end{array}$$

**Figure 12:**
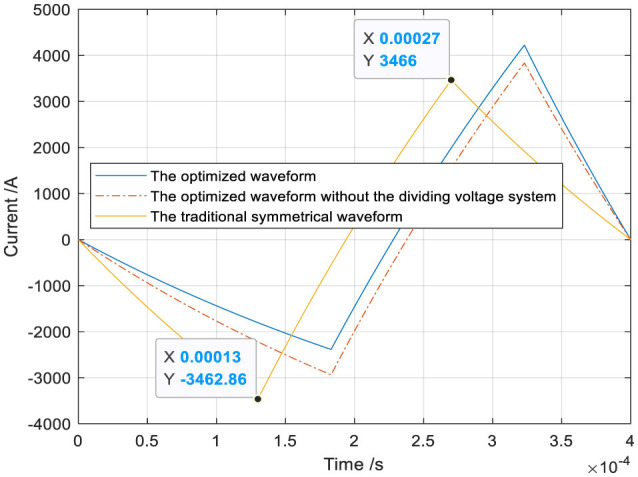
The optimized waveform with the voltage-dividing system or not and the traditional symmetrical triangular waveform.

The optimized waveform II with the voltage-dividing system and the traditional symmetrical triangular waveform are shown in Figure 12. The heat loss Q can be calculated by substituting the optimized waveform current and the traditional symmetrical triangular current into Equation 5. The ohmic loss Q (75.3169J) produced by the optimized waveform is less than that (80.3348J) produced by the traditional symmetrical waveform, and the heat loss decreases by 6.25%. The ohmic loss Q (75.3169J) produced by the optimized waveform is less than that (77.0319J) produced by the optimized waveform without the dividing system, and the heat loss decreases by 2.23%.

#### 2.3.3 Circuit model for the optimized pulse–current waveform III

**Figure 13:**
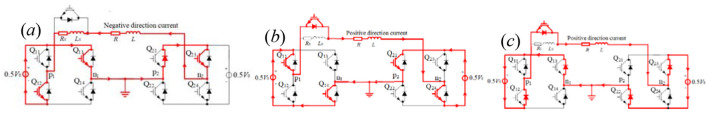
0.47*V*_0_ exerted upon the TMS coil **(a)** and ±*V*_0_ exerted upon the TMS coil **(b, c)**.

According to Equation 17, *m*_1_ = 0.47 and *m*_3_≈1 illustrate that −0.47 *V*_0_ is exerted upon the TMS coil at *t* ≤ *t*_1_ = 158 μs (shown in Figure 13a), *V*_0_ is exerted upon the TMS coil at *t*_1_ = 158μs < *t* ≤ *t*_1_ + *t*_2_ = 318 μs (shown in Figure 13b), -*V*_0_ is exerted upon the TMS coil at *t* > *t*_1_ + *t*_2_ = 318 μs (shown in Figure 13c). That is to say, for the optimized pulse–current waveform III, two modules are connected in series at the first segment, with only 1 module providing a voltage of −0.5*V*_0_ in working mode M2 and the other module providing a voltage of 0V in working mode M0, as shown in Figure 13a, where the voltage-dividing system (composed of resistance *R*_*x*_ = 3*R*/47 and inductor *L*_*x*_= 3*L*/47) is experienced by −0.03*V*_0_. At the second segment of the pulse current waveform, two modules are connected in series, with each module providing a voltage of 0.5 *V*_0_ in working mode M1, as shown in Figure 13b. At the third segment of the pulse current waveform, two modules are connected in series, with each module providing a voltage of −0.5*V*_0_ in working mode M2, as shown in Figure 13c. The voltage-dividing system is controlled by the IGBT connected in parallel with it, which is in the off state at the first segment of the triangle pulse current (*t* ≤ *t*_1_ = 158 μs) and is in the on state at the other two segments (*t* > *t*_1_ = 158 μs). At the first segment of the pulse–current, the negative current passes through the voltage-dividing system, while the negative or positive current passes through the IGBT connected in parallel with the voltage-dividing system at the second and third segments of the pulse–current. Equation 17 can be expressed by the following:


(17a) $$\begin{array}{l} i_{1}=-\frac{0.47V_{0}}{R}\left(1-e^{-\frac{R}{L}t}\right)\text{=}-\frac{0.5V_{0}}{R\text{+}R_{x}}\left(1-e^{-\frac{R+R_{x}}{L+L_{x}}t}\right),t\leq t_{1} \end{array}$$

**Figure 14:**
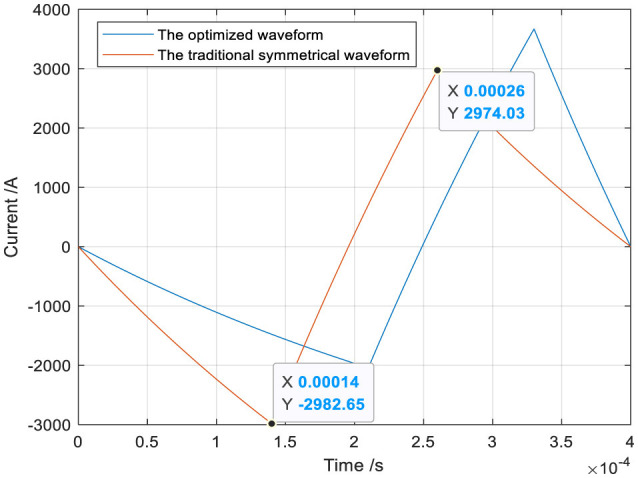
The optimized waveform with the voltage-dividing system and the traditional symmetrical triangular waveform.

The optimized waveform III with the voltage-dividing system and the traditional symmetrical triangular waveform are shown in Figure 14. The heat loss Q can be calculated by substituting the optimized waveform current III and the traditional symmetrical triangular current into Equation 5. The ohmic loss Q (100.258J) produced by the optimized waveform is less than that (104.3628J) produced by the traditional symmetrical waveform, and the heat loss decreases by 3.93%. The ohmic loss Q (100.258J) produced by the optimized waveform is less than that (100.6420J) produced by the optimized waveform without the dividing system, and the heat loss decreases by 0.38%.

In summary, we have completed the circuit design for these three optimized current waveforms. In particular, we innovatively introduced a voltage division system for the circuit designs of the latter two current waveforms. This system enables the generated current waveforms to closely match the required waveforms, not only enhancing the therapeutic effect but also reducing heat loss and noise.

The reduction in heat losses of these three optimized waveforms compared to the traditional symmetrical waveform is shown in Table 1. At least, we can conclude that the rapid decline in the third segment can reduce the residual current loss (*m*_3_ = *m*_2_ = 1), making the loss of the optimized waveform smaller than that of the traditional symmetrical waveform. The optimized waveform I achieves the greatest reduction in heat or noise (10%), and the second optimized waveform achieves a 6.25% reduction. These reductions in heat loss and noise enable patients to receive better treatment in a relatively quiet environment, thereby enhancing the therapeutic effect.

**Table 1:** The ohmic loss Q of these three optimized and traditional symmetrical waveforms.

**Waveform**	**The optimized/ J**	**The traditional/ J**	**Reduction / %**
Waveform I	53.3994	59.3301	10
Waveform II	75.3165	80.3348	6.25
Waveform III	100.258	104.3628	3.93

## 3 Results and discussion

This article discusses the principle of the approximate triangular pulse–current waveform. When the electrical energy stored in the capacitor is relatively large and the charging and discharging time is relatively short, the capacitor can be approximately regarded as a constant voltage source. The pulse–current waveform in the TMS coil is approximately triangular, and the attenuated factor is exp(–*Rt*/*L*) ≈ 1. The expressions of the pulse–current waveform are given and discussed. Next, the main influencing factors that affect this triangular pulse–current waveform were discussed. The voltage *V*_0_ exerted upon the TMS coil and its resistance *R* jointly determine the slope of the pulse–current waveform as well as its peak value. They also determine the magnitude of the induced electric field in the human brain. The slope of the pulse–current waveform increases in the positive or negative direction with |±*m*_i_*V*_0_/*L*| increasing, which causes the magnitude of the induced electric field in the human brain to increase. The membrane potential of neurons is also discussed, which is closely related to the induced electric field. When taking into account the energy loss and noise generated by the TMS coil, the pulse–current waveform was optimized, and its expressions were derived and given. Under the given conditions, this pulse–current waveform optimization is transformed into determining the slope (d*i*_1_/d*t*) of the first segment of the pulse–current waveform, mainly *m*_1_, and its pulse width *t*_1_ when the ohmic loss and clicking noise of the TMS coil are at their lowest. This article obtained three relatively typical optimized waveforms using theoretical calculations and the particle swarm optimization algorithm, which could provide a theoretical basis and technical reference for clinical practice in pulse–current waveform optimization and future circuit design.

Finally, this article presents a novel and applicable circuit model that can generate various required triangle pulse–current waveforms to reduce the TMS coil ohmic loss and clicking noise. Unlike the traditional TMS circuit, in this study, only one power source is required to provide a constant voltage, which can significantly reduce the complexity of the circuit power supply. Using the multi-module stacking and the voltage-dividing system, it is possible to supply almost any voltage level to the TMS coil, achieving the minimum energy consumption and low noise (clicking noise) for the optimized pulse–current waveform under the given conditions.

Compared with the recently presented multi-module stacking (Zeng et al., 2022; Nilsson and Riedel, 2010; Fang et al., 2024), the presented novel circuit system does not use high-frequency switches to obtain the ultrashort pulses for achieving the required pulse–current waveform shape. For the recently presented pulse–current waveform in Zeng et al. (2022), Nilsson and Riedel (2010), and Fang et al. (2024), the shorter the time of the ultrashort pulse is, the closer the pulsed current waveform formed by the ultrashort pulse current will be to the required waveform. However, if the time of the ultrashort pulse is too long or its period is too long, there will be a significant deviation between the generated pulsed-current waveform and the required waveform. The multi-module stacking connection can only provide multiple discrete voltages with a multiple relationship and reduce the usage time and frequency of IGBTs within each module, which is perhaps beneficial for prolonging the service life of the devices, but it cannot reduce the switching frequency. Additionally, the multi-module cascading will increase the complexity of the TMS circuit system. In this study, the voltage-division system can effectively reduce the number of multi-module stackings, resulting in continuous variable voltage levels.

It is known that the triangle pulse–current waveform can provide an approximate rectangular induced electric field (ARIEF) in the human brain. Generally, the amplitude of the ARIEF is defined by the slope (*di*_2_/*dt*) of the second segment of the pulse–current waveform, as is demonstrated by Figures 1c, d. The TMS clinic practice determines the second segment (*di*_2_/*dt*, *t*_2_) of the optimized pulse–current waveform. In order to reduce the TMS ohmic loss and its clicking noise, the third segment of the pulse–current waveform must decline as rapidly as possible. Therefore, optimizing the pulse–current waveform mainly involves determining the slope and duration of the first segment, of course, the whole period (*T*) of the pulse–current waveform, *t*_2_, the power supply (*V*_0_) and the TMS coil system (*R, L*) is firstly determined by the clinical practice and its corresponding conditions. Therefore, after finishing analyzing the sensitivity of the pulse–current waveform, the study presents three relatively typical optimized pulse–current waveforms according to the duration of the second segment or the requirements of the required TMS practices.

For the first optimized pulse–current waveform, when its period *T* = 400 μs and the duration of the second segment *t*_2_ = 120 μs, the slope of the first segment and its duration *t*_1_ are obtained by numerical calculation and the particle swarm optimization algorithm. *m*_1_ = 0.25 illustrates that the maximum slope values (*m*_2_*V*_0_/*L* =*m*_3_*V*_0_/*L* =*V*_0_/*L*) of the second and third segments are exactly four times the slope of the first segment. It is precisely because of this case (*m*_1_ = 0.25, *m*_2_ = *m*_3_ = 1) that a circuit model with exactly four modules connected in series can be designed. However, *m*_1_ will decrease when the period *T* increases or *t*_2_ decreases. For example, *m*_1_ = 0.1 when *t*_2_ = 80 μs. *m*_1_ = 0.1 demonstrates that a circuit model with exactly 10 modules connected in series should be designed. *m*_1_ will increase when *t*_2_ increases.

For the second optimized pulse–current waveform, when *T* = 400 μs and *t*_2_ = 140 μs, *t*_1_ = 183 μs and *m*_1_ = 0.325 can be found by numerical calculation and the particle swarm optimization algorithm. *m*_1_ = 0.325 illustrates that a circuit model with 13 modules connected in series will provide 325 V (V_0_ = 1,000V) at the first segment, each module providing 25 V. Of course, the circuit model with 40 modules connected in series will provide 1,000 V at the second segment and −1,000 V at the third segment. The TMS circuit system is very bulky and complex, with an equally intricate control system and PWM compiler. Additionally, the energy loss in the circuit system is substantial. In this study, *m*_1_ = 0.4 is set up so that a series connection of 5 modules can be used for obtaining ±1,000 V at the second and third segments, and a series connection of 2 modules can be used for obtaining 400 V at the first segment. In the first segment, the voltage-dividing system is used to divide the voltage (400 V) by 75 V; thus, the voltage finally applied to the coil system is 325 V. The voltage-dividing system, composed of the resistance *R*_*x*_ = 3*R*/13 and inductor *L*_*x*_= 3*L*/13, is simple and easily designed. Moreover, the voltage-dividing system can be controlled by an IGBT (only in the off state at the first segment) connected in parallel with it, which will not additionally increase the energy loss and clicking noise of the TMS coil.

For the third pulse–current waveform III, when *T* = 400 μs and *t*_2_ = 160 μs, *t*_1_ = 158 μs, and *m*_1_ = 0.47 can be found by numerical calculation and the particle swarm optimization algorithm. Similarly, *m*_1_ = 0.5 is set up first. *m*_1_ = 0.5 illustrates that a circuit model with one module will provide 500 V at the first segment, and two modules connected in series will provide ±1,000 V at the second and third segments, each module providing 500 V. Secondly, the voltage-dividing system is designed to divide the voltage (500 V) by 30 V at the first segment of the pulse–current waveform. The energy consumption of the voltage-dividing system is negligible and can be safely ignored. Otherwise, according to the principle of multi-module cascading, if the voltage-dividing system is not introduced, 100 modules need to be connected in series to supply a voltage of ±1,000 V to the TMS coil at the second and third segments of the pulse–current waveform, and 47 modules need to be connected in series to supply a voltage of 470 V to the TMS coil at the first segment.

In summary, this article introduces a voltage-dividing system for generating optimized pulse–current triangular waveforms. The voltage-dividing system can provide continuously variable voltage levels, addressing the gap between discrete voltage levels. It is more applicable in practical scenarios compared to those discrete voltage levels. Second, the circuit system can avoid the use of high-frequency pulses by employing a low-frequency PWM controller, which can prevent the devices from working to fatigue and extend their service life. Finally, both the theoretical analysis and expressions of the optimized waveforms, as well as the voltage-dividing model, have been provided, and they are relatively complete and comprehensive. It is foreseeable that these results and expressions will be proved correct in future experiments and practices.

In this study, the introduced voltage-dividing system does not impact the thermal loss or clicking noise of the TMS coil, but it does consume the electric power of the power supply, resulting in lower efficiency. Future research will focus on experimental verification.

The above results and conclusions have been proven to be correct theoretically and are consistent with the simulations in MATLAB Simulink, indicating their validity. If practical experiments are carried out, those results should conform to the expected theoretical analysis and simulations when the conditions are met. However, it will take a considerable amount of time to conduct and complete these validations because the designs of the power supply system, measurement system, water cooling system, and other components are somewhat complex. In the future, we will conduct experimental studies to further prove these findings, providing a foundation and basis for the clinical applications of this novel technology.

## Data Availability

The original contributions presented in the study are included in the article/supplementary material, further inquiries can be directed to the corresponding author.
